# Tea polyphenol EGCG inhibited colorectal-cancer-cell proliferation and migration via downregulation of STAT3 

**DOI:** 10.1093/gastro/goaa072

**Published:** 2020-12-03

**Authors:** Ke-Wang Luo, Jun Xia, Bao-Hui Cheng, Han-Chao Gao, Li-Wu Fu, Xin-Le Luo

**Affiliations:** 1 Key Laboratory, People’s Hospital of Longhua, Shenzhen, Guangdong, P. R. China; 2 Key Laboratory of Medical Programming Technology, Shenzhen Second People’s Hospital, The First Affiliated Hospital of Shenzhen University, Shenzhen, Guangdong, P. R. China; 3 Shenzhen Key Laboratory of ENT, Longgang ENT Hospital and Institute of ENT, Shenzhen, Guangdong, P. R. China; 4 Department of Nephrology, Shenzhen Longhua District Central Hospital, Affiliated Central Hospital of Shenzhen Longhua District, Guangdong Medical University, Shenzhen, Guangdong, P. R. China; 5 State Key Laboratory of Oncology in South China, Collaborative Innovation Center for Cancer Medicine, Guangdong Esophageal Cancer Institute, Sun Yat-sen University Cancer Center, Guangzhou, Guangdong, P. R. China

**Keywords:** EGCG, colon cancer, STAT3, SW480, SW620, LS411N

## Abstract

**Background:**

Green tea is a popular beverage worldwide and epigallocatechin-3-gallate (EGCG) is the most bioactive polyphenol in green tea. Our study aims to investigate the anti-proliferation and anti-migration effects of EGCG against colorectal-cancer SW480, SW620, and LS411N cells, and elucidate the underlying mechanism.

**Methods:**

The *in vitro* anti-proliferation and anti-migration effects of EGCG against colon-cancer cells were evaluated using MTT, scratch-wound-healing, and transwell-migration assays. The effects of EGCG on apoptosis were assessed by Annexin V-FITC/PI double staining and JC-1 staining. Besides, Western blotting was employed to detect the protein-expression level and elucidate the underlying pathways. Real-time qPCR and dual-luciferase reporter assay were adopted to determine the mRNA level and promoter activity.

**Results:**

Our results demonstrated that treatment with EGCG resulted in significant inhibition of cell proliferation by the induction of apoptosis. EGCG also inhibited SW480 cell migration in a dose-dependent manner as assessed by wound-healing and transwell-migration assays. Western blot confirmed that EGCG induced apoptosis by the activation of Caspase-3 and PARP. In addition, both STAT3 and phosphorylated STAT3 (p-STAT3) were downregulated significantly by EGCG in three selected colorectal-cancer cell lines. EGCG treatment also resulted in a significant decrease in Bcl-2, MCL-1, and Vimentin, and an increase in E-cadherin. When STAT3 was inhibited, EGCG showed no obvious effect on cell proliferation and migration. Further investigation by luciferase-reporter-activity assay showed that EGCG suppressed the promoter activity of STAT3 and downregulated the transcription of STAT3.

**Conclusion:**

Our study presents evidence on the anti-proliferation and anti-migration effects of EGCG against colorectal-cancer SW480, SW620, and LS411N cells by downregulating the expression of STAT3 and suggests that EGCG could be an effective and natural supplement for colon-cancer treatment.

## Introduction

Despite significant advances in cancer research, cancer remains a worldwide health problem, accounting for an estimated 9.6 million deaths in 2018. Colon cancer is one of the most malignant types of cancer, which ranks second in the world for cancer deaths and caused 880,000 deaths in 2018. Worldwide, colorectal cancer is the third most common type of cancer in men and second most common in women, accounting for an estimated 1.85 million new cases in 2018 [1]. Therefore, colon cancer still takes a tremendous toll and novel therapeutic strategies and more effective agents for advanced disease are still needed.

Green tea, one of the most well-known beverages worldwide, is obtained from the dried leaves of the plant *Camellia sinensis*. Green tea contains >200 bioactive compounds, including tea polyphenols, caffeine, theanine, vitamins, and minerals. The largest and most active group of tea components is tea polyphenols, including epicatechin, epicatechin gallate, epigallocatechin, and epigallocatechin gallate (EGCG) [2]. In our previous studies, high-performance liquid-chromatography analysis showed that EGCG was the most abundant ingredient in green-tea water extracts [3]. EGCG is demonstrated to have various biological activities, including anti-obesity, anti-oxidation, and anticancer effects [2]. Especially regarding anticancer effects, a study stated that a number of pieced of literature have reported the efficacy of EGCG in the prevention and treatment of different kinds of cancers in clinical, *in vivo*, and *in vitro* studies [4]. A clinical study indicated that EGCG could help in reducing the incidence of patients with pre-adenocarcinoma [5]. In an animal study, treatment with EGCG resulted in the inhibition of tumorigenesis in gastrointestinal cancer, lung cancer, and prostate cancer [6]. Our previous study also demonstrated that EGCG was effective in the inhibition of bladder-cancer-cell proliferation and migration in mice bearing SW780 tumors by the downregulation of NF-κB and MMP-9 [7]. Besides, EGCG was also shown to be effective in cell proliferation or apoptosis and regulating cancer stem cells by interacting directly with Pin1, TGFR-II, NF-κB, and metalloproteinases (mainly MMP2 and MMP9) [8]. Treatment with EGCG resulted in significant induction of apoptosis in chronic myeloid leukemia, by regulating the JAK2/STAT3/AKT-signaling pathway [9]. Recently, varieties of studies have investigated the effects of EGCG in colon cancer. An *in vivo* study showed that peracetylated-EGCG suppressed colitis and colon tumorigenesis in mice [10]. EGCG in nanoparticle form was effective at enhancing the anti-colon-cancer efficacy of 5-fluorouracil with a better bioavailability and longer circulation time *in vivo* [11]. In addition, *in vitro* studies have demonstrated that EGCG inhibited cell growth and migration in colon-cancer SW620, Caco-2, and SW480 cells, and downregulated EGFR-expression levels [12–14]. However, few reports have evaluated the anticancer effects and underlying mechanisms of EGCG in primary colon-cancer SW480 cells and in metastatic colon-cancer SW620 and LS411N cell lines.

During the process of cancer propagation, signaling and transcriptional activation factor 3 (STAT3) play an important role. STAT3, a membrane receptor-mediated nuclear transcription factor ubiquitously expressed in cells and tissues, plays a critical role in the regulation of cell differentiation, proliferation, and metastasis [15]. Usually, STAT3 exists in the cytoplasm in a dimerized form with peptide ligands. Once STAT3 is activated by Janus kinase (JAK), it will convert into phosphorylated STAT3 (p-STAT3), thereby triggering dimerization, forming STAT3 dimers in the cytoplasm, and translocating from the cytoplasm into the nucleus. Thus, STAT3 activates the transcription of target genes downstream, promotes cell-cycle progression, and inhibits apoptosis and angiogenesis by regulating Bcl-2 family members, MCL-1, etc. [16]. It is reported that cytokine receptors, signaling pathways, and epigenetic regulators, could induce STAT3 expression [17]. EGCG was demonstrated to be effective in downregulating the expression of STAT3 in breast-cancer stem-cell phenotype MDA-MB-231 cells and MCF7 cells [18]. Besides, EGCG was effective at eliminating the stem-like properties and enhancing the chemosensitivity in nasopharyngeal carcinoma through the attenuation of STAT3 activation [19]. However, few reports have investigated the efficacy of EGCG on STAT3 expression and clarified the underlying mechanisms against primary and metastatic colon-cancer cells. Our study showed that EGCG inhibited colon-cancer-cell proliferation and migration in both primary SW480 cells and metastatic SW620 and LS411N cell lines by suppressing the promoter activity of STAT3 and downregulating the transcription of STAT3.

In the present study, we investigated the anti-proliferation and anti-migration effects of tea polyphenol EGCG in colorectal-cancer SW480, SW620, and LS411N cell lines. Also, the role of EGCG in different mechanisms of action was be discussed. Here, we assessed the apoptosis-induction and anti-migration abilities of EGCG *in vitro* and then further evaluated the protein-expression level, especially STAT3 and p-STAT3 expression, after treatment with EGCG. In addition, the efficacy of EGCG on STAT3 messenger RNA (mRNA) expression and STAT3 promoter activity was also evaluated.

## Material and methods

### Cells and reagents

SW480, SW620, and LS411N colorectal-cancer cells were obtained from American Type Culture Collection (Manassas, VA, USA) and cultured in Dulbecco’s modified eagle medium (DMEM; Gibco; Carlsbad, CA, USA) containing 10% v/v fetal bovine serum (FBS) and 1% penicillin-streptomycin (Sigma; St. Louis, MO, USA) at 37°C in a 5% CO_2_ humidified incubator. 3-(4,5-Dimethylthiazol-2-yl)-2,5-diphenyltetrazolium bromide (MTT) was obtained from Sigma. An Annexin V-fluorescein isothiocyanate/propidium iodide (Annexin V-FITC/PI) kit was purchased from BD Pharmingen (Franklin Lakes, NJ, USA). 5,5′,6,6′-Tetrachloro-1,1′,3,3′-tetraethylbenzimidazolylcarbocyanine iodide (JC-1) was obtained from Molecular Probes, Inc. (Eugene, OR, USA). Transwell plates were obtained from Corning Inc. (Corning, NY, USA). Caspase-3, PARP (9542S), Bcl-2 (2870S), STAT3 (9139S), p-STAT3 (9145S), Bim (2819S), Bak (6947S), MCL-1 (4572S), E-cadherin (3195S), Vimentin (3932S), and β-actin were purchased from Cell Signaling Technology (Boston, MA, USA). Stattic, the STAT3 inhibitor, was obtained from Selleck Chemicals (Houston, TX, USA).

### Cell-viability assay

SW480, SW620, and LS411N cells (1 × 10^4^/well) were seeded in 96-well plates (Corning Inc.) and incubated with different concentrations of EGCG for 24 h. Following incubation, 30 µL of 5 mg/mL MTT solution was added to each well and the plate was incubated at 37°C for another 4 h. Then, the medium was discarded and 150 µL of dimethylsulfoxide (DMSO; Sigma) was added to dissolve the formazan crystals. The absorbance of each sample was read at 540 nm using a microplate reader (Thermo Multiskan GO; Waltham, MA, USA).

### Annexin V-FITC/PI double staining

After treatment with EGCG, cells were collected and washed with ice-cold phosphate buffer saline (PBS) and then stained in 300 μL solution containing Annexin V-FITC and PI for 15 min in the dark at room temperature. Subsequently, the fluorescent signal was detected by flow cytometry (Becton Dickinson; Franklin Lakes, NJ, USA). The positioning of quadrants on Annexin V-FITC/PI plots was performed to distinguish living cells (FITC−/PI−), early apoptotic cells (FITC+/PI−), and late apoptotic or necrotic cells (FITC+/PI+).

### Detection of mitochondrial-membrane potential change

The mitochondrial-membrane potential was analysed by JC-1 staining. Cells (3 × 10^5^/well) were seeded in six-well plates and incubated with EGCG for 24 h. After treatment, the cells were harvested, washed with PBS, and resuspended in JC-1 staining solution (10 µM) at 37°C for 15 min. Subsequently, the cells were examined using a FACSCanto flow cytometer (Becton Dickinson). Data analysis was performed using the WinMDI 2.9 (BD; San Diego, CA, USA) software.

### Wound-healing assay

Cells (3 × 10^5^/well) were seeded into a six-well culture plate and grown to 100% confluence in full-growth medium. After being starved in serum-free medium overnight, cells were scraped with a plastic pipette tip to produce a clean-wound area across the center of the well. Then, the cell debris was washed away using PBS and cells were allowed to migrate into DMEM full-growth medium for the indicated time. The wound widths were photographed by an Olympus IX73 inverted microscope (Olympus; Tokyo, Japan) at 0, 24, and 48 h.

### Transwell-migration assay

Cells (2 × 10^4^/well) were added into transwell chambers, together with 100 µL medium containing various concentrations of EGCG (with 1% v/v FBS; Gibco). Then, 500 µL complete DMEM medium (with 10% v/v FBS) served as chemoattractant media and was placed below the cell-permeable membrane. After being incubated at 37°C for 24 h, cells were fixed with methanol and stained with 0.1% crystal violet. Stained filters were photographed by microscope (Olympus IX73). The migrated cells were quantified by manual counting. The cell migration was expressed as the relative migration rate.

### Western blot analysis

After being treated with EGCG, cells were lysed in lysis buffer for 30 min. Then, the lysate was boiled and denatured protein samples (20 µg) were loaded into wells in 10% sodium dodecyl sulfate (SDS)-polyacrylamide gel. Following electrophoretic separation, the proteins were transferred to polyvinylidene difluoride (PVDF) membrane (Millipore; Burlington, MA, USA). After being blocked using 10% non-fat milk, the membranes were washed using PBS containing 0.1% (v/v) Tween-20 (PBS-T) and then incubated with primary antibodies (dilution at 1:1,000) for 2 h at 4°C. After being washed with PBS-T, the membrane was incubated with secondary antibodies conjugated with horseradish peroxidase for 1 h. Finally, visualization of protein bands was performed using the enhanced chemiluminescence (ECL) substrate reagent kit (GE Healthcare; Stockholm, Sweden) on a Gel Doc XR imaging system (Bio-RAD; Hercules, CA, USA).

### Real-time quantitative PCR

After being treated with EGCG, the total RNA was extracted from the SW480 cells using TRIzol reagent (Invitrogen; Carlsbad, CA, USA). The mRNA level of STAT3 was determined by real-time quantitative PCR (qPCR) following the instructions. The GAPDH mRNA was used as an internal control to normalize the amount of mRNA in each sample. Our preliminary results have shown that GAPDH is suitable for normalization purposes. The primers were designed as follows: GAPDH forward: 5′-AAG GTG AAG GTC GGA GTC AAC-3′, reverse: 5′-GGG GTC ATT GAT GGC AAC AAT A-3′; STAT3 forward: 5′- ACC TTT GAG ACC GAG GTG TA-3′, reverse: 5′- CAC CAG GTC CCA AGA GTT TC-3′. The reactions were performed in triplicate using the ABI VII7 Fluorescent Quantitative PCR System (Thermo; Waltham, MA, USA). The average value in each triplicate was used to calculate the relative amount of STAT3 mRNA using the comparative ΔCt method.

### Luciferase-reporter-activity assay

The whole STAT3 promoter region (nucleotides –1,000 to +1 bp to the translation initiation site) was synthesized from SW480-cell-line-genomic DNA and inserted into the plasmid pGL3 (Promega; Madison, WI, USA). Then, SW480 cells were transfected with 1.2 mg of luciferase-reporter vector containing a STAT3 promoter sequence (pLG3-STAT3) using Lipofectamine 2000 (Invitrogen) and a pLG3 empty vector (pLG3-basic) was used as a negative control. Following transfection, EGCG (50 µg/mL) and Stattic (1 µM) were added to the SW480 cells and incubated for 24 h. After incubation, the cells were washed and lysed according to the Dual Luciferase Assay kit (Promega) and the luciferase activities were measured using a FLUOstar Galaxy plate reader (Promega; Sunnyvale, CA, USA).

### Statistical analysis

Each experiment was performed three times and all data in the graph were expressed as mean ± standard deviation (SD). Statistical differences were calculated using the one-way analysis of variance, with *P *<* *0.05 considered as statistically significant.

## Results

### EGCG inhibited SW480-, SW620-, and LS411N-cell proliferation

**Figure 1.**
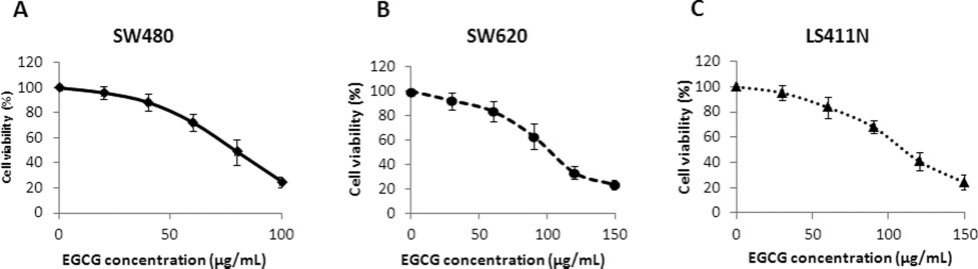
Cytotoxicity of EGCG on colorectal-cancer SW480 (A), SW620 (B), and LS411N (C) cells, after 24-h incubation. Data were expressed as mean ± SD.

Treatment with EGCG for 24 h resulted in the inhibition of cell proliferation in a dose-dependent manner. As shown in Figure 1, EGCG inhibited the growth of SW480, SW620, and LS411N cells with an IC_50_ of 74.6, 99.4, and 112.1 μg/mL at 24 h, respectively. Besides, the results showed that SW480 cells were more sensitive to EGCG than SW620 and LS411N cells (Figure 1).

### EGCG induced apoptosis in SW480, SW620, and LS411N cells

**Figure 2.**
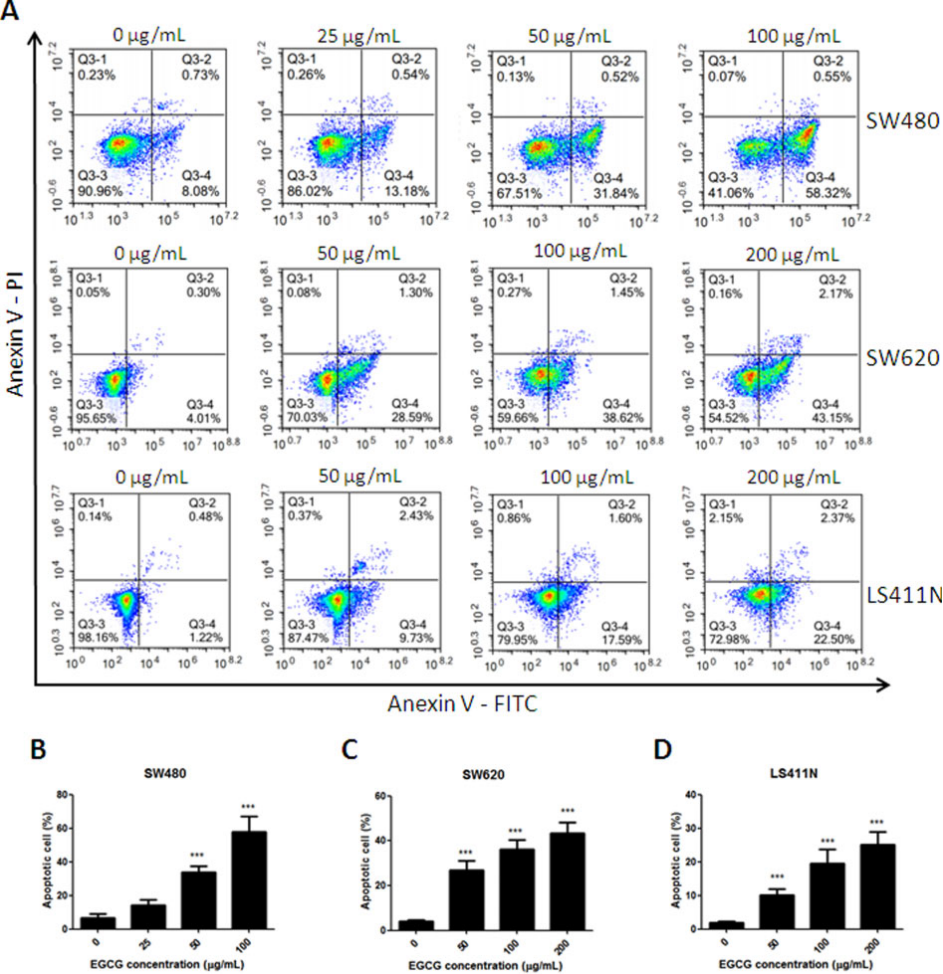
Induction of apoptosis on SW480, SW620, and LS411N cells by EGCG. (A) Flow-cytometry images. (B)–(D) Quantitative analysis of the percentage of apoptotic cells of EGCG on SW480 (B), SW620 (C), and LS411N (D) cells, after 24-h incubation. The percentage of total apoptotic cells was defined as the sum of early and late apoptotic cells. **P <* *0.01 and ***P <* *0.001, as compared with untreated control.

Annexin V-FITC/PI staining showed that, when SW480, SW620, and LS411N cells were incubated with increasing doses of EGCG from 0 to 200 μg/mL, the rates of cell apoptosis were increased in a dose-dependent manner (Figure 2A). The percentage of apoptotic cells upon treatment with 25, 50, and 100 μg/mL of EGCG in SW480 cells was found to be 13.62%, 32.36%, and 58.87%, respectively, after 24-h incubation (Figure 2B). Similar results were also found in SW620 and LS411N cells: EGCG induced SW620- and LS411N-cell apoptosis in a dose-dependent manner and significant differences were shown between EGCG (50, 100, 200 μg/mL) and control (Figure 2C and D).

### EGCG induced disruption of mitochondrial-membrane potential

**Figure 3.**
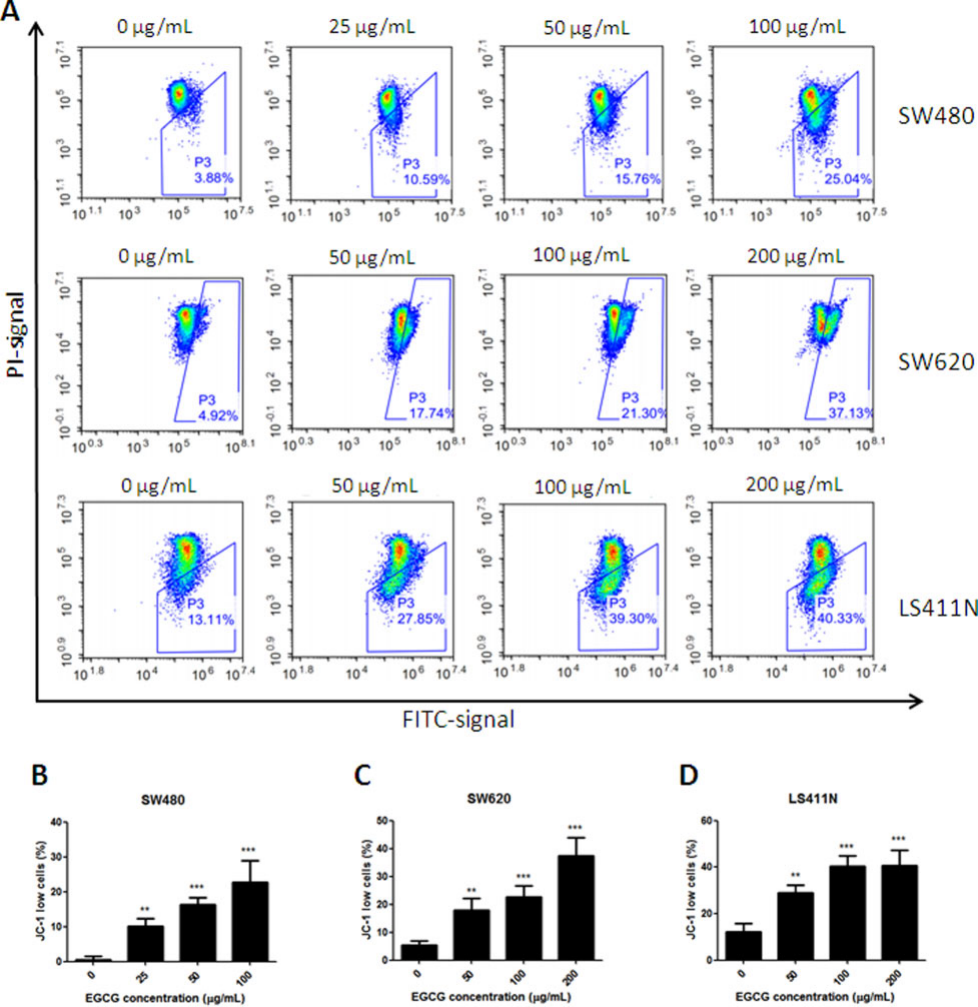
Collapse of the mitochondrial-membrane potential induced by EGCG. (A) Flow-cytometry images. (B)–(D) Quantitative analysis of the percentage of JC-1 low cells in SW480 (B), SW620 (C), and LS411N (D) cell lines, after treatment with EGCG. The percentages represent cells with a depolarized mitochondrial membrane. **P <* *0.01 and ***P <* *0.001, as compared with untreated control.

As shown in Figure 3, the disruption of the mitochondrial-membrane potential induced by EGCG was dose-dependent. Upon EGCG treatment, the percentage of SW480 cells with a low level of JC-1 increased to 10.59%, 15.76%, and 25.04%, at 25, 50, and 100 µg/mL, respectively. Consistent results were shown in metastatic colorectal-cancer SW620 and LS411N cells. EGCG induced disruption of the mitochondrial-membrane potential in SW620 and LS411N cells, and the percentage of JC-1 low cells reached 17.74%, 21.3%, and 37.13% in SW620 cells, and 27.85%, 39.3%, and 40.33% in LS411N cells, in the presence of 50, 100, and 200 μg/mL EGCG, respectively. Among the three selected colorectal-cancer cell lines, the SW480 cell line was the most sensitive to EGCG on mitochondrial-membrane-potential disruption.

### EGCG inhibited SW480-cell migration

**Figure 4.**
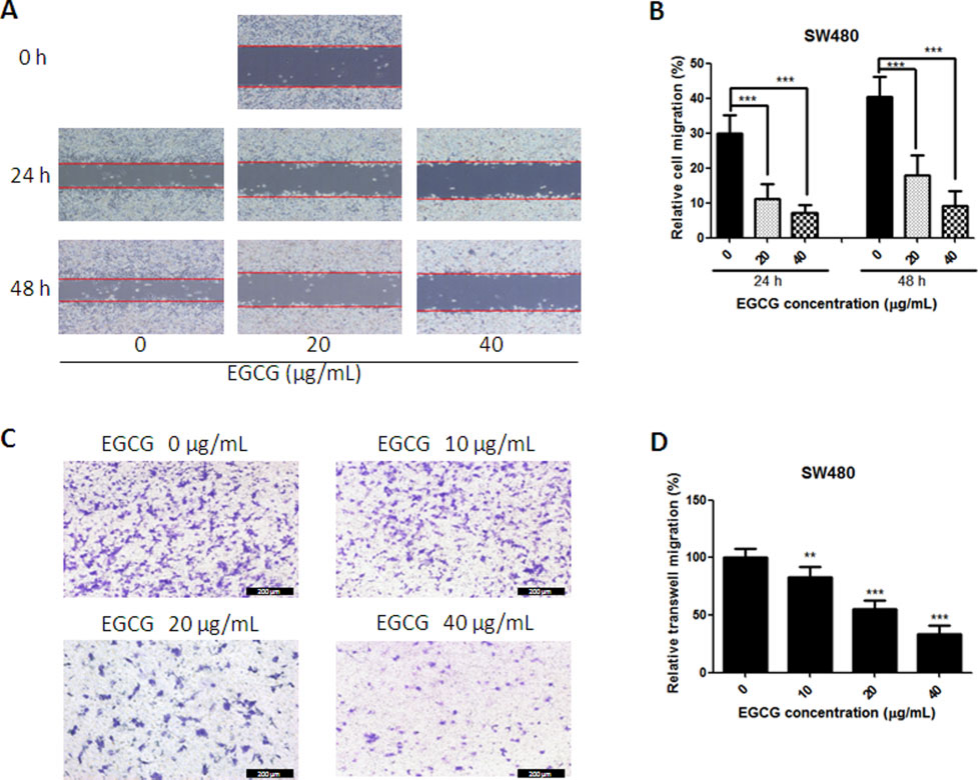
Effect of EGCG on SW480-cell-migration activities. (A) Representative images of the wounded-cell monolayers of SW480 cells. (B) Quantitative analysis of the anti-migration activity of EGCG after 24- and 48-h incubation. Data are expressed as the percentage of the relative gap distance from baseline cultures without treatment. (C) Representative images of the stained SW480 cells. (D) Quantitative analysis of the anti-transwell-migration activity of EGCG. Data are presented as mean + SD (*n *=* *3). **P <* *0.01 and ***P <* *0.001, as compared with untreated control.

To determine the efficacy of EGCG against cancer-cell metastasis *in vitro*, the wound-healing and transwell-migration assays were implemented. Since the SW480 cell line was the most sensitive to EGCG regarding cell proliferation and apoptosis induction among the three selected colorectal-cancer cell lines, the SW480 cell line was then chosen for the detection of migration. As shown in Figure 4A, EGCG inhibited SW480-cell migration since 20 μg/mL after 24-h incubation, with inhibition rates of >55%. The inhibition was enlarged when the EGCG concentration was increased and the treatment time prolonged (Figure 4B). Also, the result from the transwell-migration assay was in line with the data from the scratch assay. In Figure 4C, EGCG inhibited SW480-cell migration efficiently with the increase in EGCG concentration. In the presence of 10, 20, and 40 μg/mL, EGCG inhibited SW480-cell migration significantly by 16.7%, 44.8%, and 66.5%, respectively (Figure 4D).

### EGCG regulated the protein expressions

**Figure 5.**
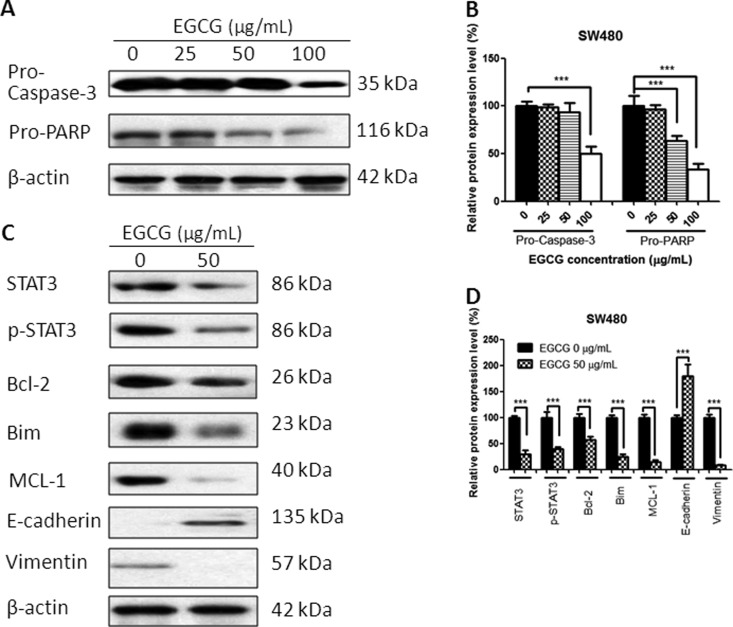
Effect of EGCG on protein expression in SW480 cells. (A) Representative images of Western blot of Caspase-3 and PARP in SW480 cells after treatment with EGCG. (B) Statistical analysis of Caspase-3, PARP in SW480 cells after EGCG treatment. (C) Representative images of Western blot of STAT3, Bcl-2, Bim, MCL-1, E-cadherin, and Vimentin after treatment with EGCG. (D) Statistical analysis of STAT3, p-STAT3, Bcl-2, Bim, MCL-1, E-cadherin, and Vimentin in SW480 cells after EGCG treatment. Data are shown as mean + SD (*n *=* *3). **P <* *0.01 and ***P <* *0.001, as compared with untreated control.

**Figure 6.**
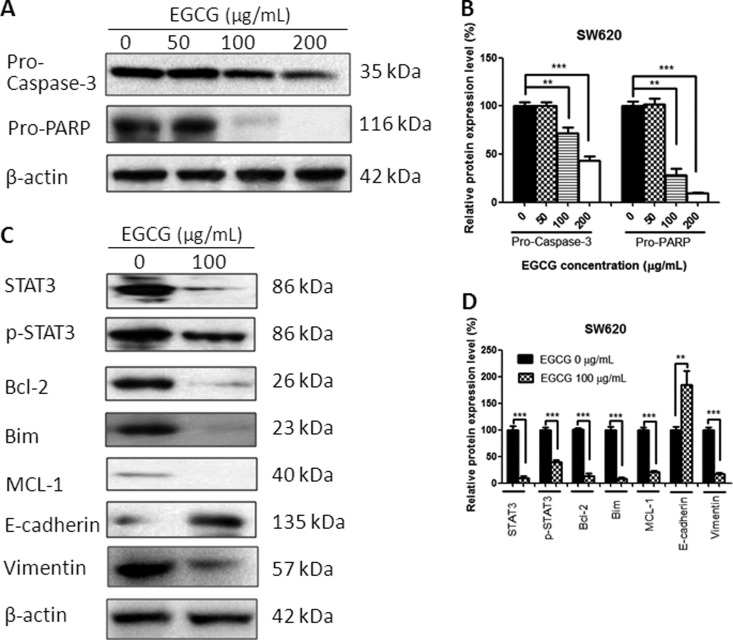
Effect of EGCG on protein expression in SW620 cells. (A) Representative images of Western blot of Caspase-3 and PARP after treatment with EGCG. (B) Statistical analysis of Caspase-3, PARP in SW620 cells after EGCG treatment. (C) Representative images of Western blot of STAT3, Bcl-2, Bim, MCL-1, E-cadherin, and Vimentin after treatment with EGCG. (D) Statistical analysis of STAT3, p-STAT3, Bcl-2, Bim, MCL-1, E-cadherin, and Vimentin in SW620 cells after EGCG treatment. Data are shown as mean + SD (*n *=* *3). **P <* *0.01 and ***P <* *0.001, as compared with untreated control.

**Figure 7.**
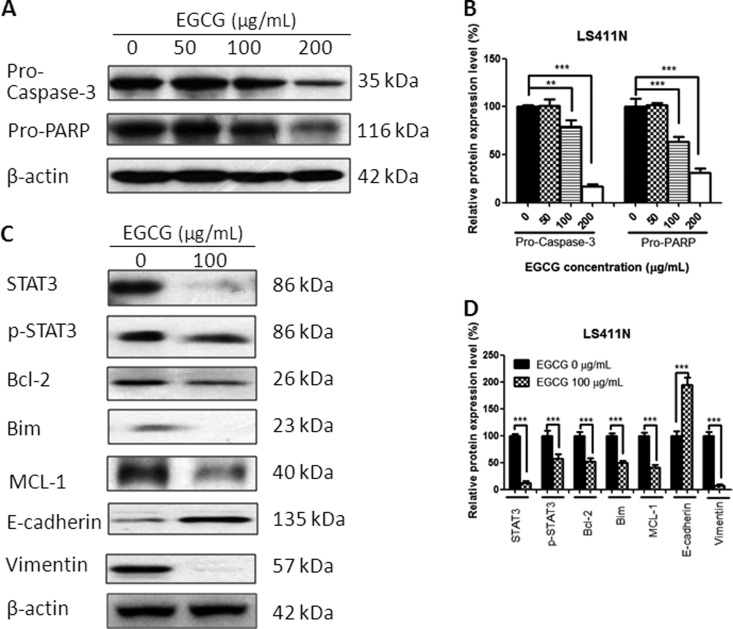
Effect of EGCG on protein expression in LS411N cells. (A) Representative images of Western blot of proteins after treated with EGCG. (B) Statistical analysis of Caspase-3, PARP in LS411N cells after EGCG treatment. (C) Representative images of Western blot of STAT3, Bcl-2, Bim, MCL-1, E-cadherin, and Vimentin after treatment with EGCG. (D) Statistical analysis of STAT3, p-STAT3, Bcl-2, Bim, MCL-1, E-cadherin, and Vimentin in LS411N cells after EGCG treatment. Data are shown as mean + SD (*n *=* *3). **P <* *0.01 and ***P <* *0.001, as compared with untreated control.

Treatment with EGCG for 24 h resulted in a change in protein expression. The EGCG-treated colorectal-cancer cells demonstrated the cleavage of protein Caspase-3 and PARP in a dose-dependent manner, indicating the apoptosis-induction effects of EGCG in SW480, SW620, and LS411N cells (Figures 5–7A and B). Besides, EGCG downregulated significantly STAT3 and p-STAT3 expression in these three colorectal-cancer cell lines. EGCG also decreased the expression of Bcl-2, Bim, and MCL-1. In addition, E-cadherin was upregulated and Vimentin was downregulated after EGCG treatment (Figures 5–7C and D), indicating the inhibitory effect of EGCG on cell migration.

### EGCG showed no obvious effect on SW480-cell proliferation and migration when STAT3 was inhibited

**Figure 8.**
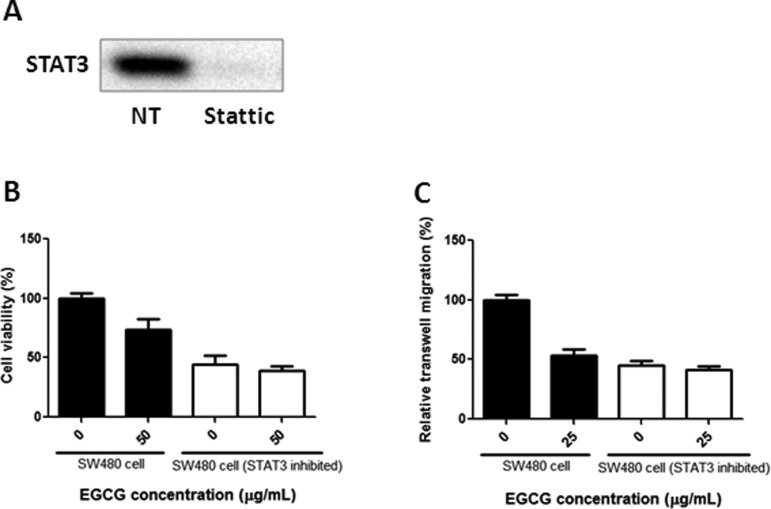
Anti-proliferation and anti-migration effects of EGCG in normal and STAT3-inhibited SW480 cells. (A) STAT3 was inhibited in SW480 cells after treatment with Stattic. Anti-proliferation (B) and anti-migration (C) effects of EGCG on normal and STAT3-inhibited SW480 cells. Data are expressed as mean + SD (*n *=* *3).

In order to confirm the important role of STAT3 in EGCG-induced proliferation and migration inhibition, 2 μM Stattic was added to SW480 cells and then the cells were collected for MTT and transwell assays. As shown in Figure 8A, the STAT3 was totally suppressed when the inhibitor Stattic was added. In Figure 8B, EGCG inhibited cell proliferation in normal SW480 cells but showed no obvious effect on STAT3-inhibited SW480 cells. A similar result was also present in transwell assays that EGCG treatment resulted in no significant difference in STAT3-inhibited SW480 cells compared with untreated controls (Figure 8C).

### EGCG suppressed the transcription of STAT3 by inhibiting the promoter activity

**Figure 9.**
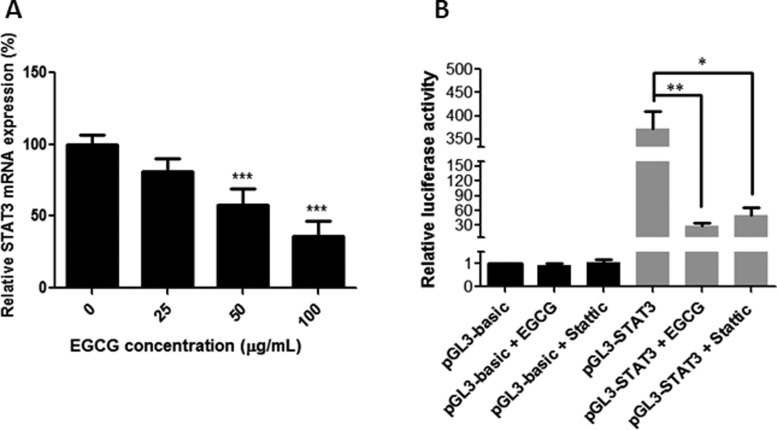
Effect of EGCG on STAT3 mRNA expression and truncation analysis of the STAT3 promoter with EGCG. (A) The relative mRNA-expression level of STAT3 was evaluated using real-time qPCR. (B) The relative luciferase activity of truncated STAT3 promoter was detected using the Dual-Luciferase Reporter Assay System. SW480 cells were transiently transfected using the truncated STAT3-promoter reporters followed by treatment with EGCG for 24 h. After that, the cells were lysed and the luciferase activity was measured. Data are shown as mean + SD (*n *=* *3). *P <* *0.05, **P <* *0.01, and ***P <* *0.001, as compared with untreated control.

In order to confirm whether EGCG was effective at regulating the mRNA-expression level of STAT3, real-time qPCR was performed. As shown in Figure 9A, EGCG treatment resulted in a significant decrease in STAT3 expression at doses of 50 and 100 μM in SW480 cells. Besides, a dual-luciferase reporter assay was adopted to assess the efficacy of EGCG on the promoter activity of STAT3. The promoter sequence of STAT3 was cloned into plasmid pGL3 and transfected into SW480 cells. Then, the transfected cells were incubated with EGCG or Stattic for 24 h and the luciferase activities were determined by dual-luciferase reporter assay. As shown in Figure 9B, EGCG remarkably decreased the luciferase activities of the reporters in the STAT3 promoter region and EGCG (50 μg/mL) even showed better effects in the downregulation of luciferase activity when compared with the STAT3 inhibitor Stattic (1 μM). The results suggest that the suppression effect of EGCG on the STAT3 promoter may be responsible for the downregulation of STAT3 transcription.

## Discussion

Green tea is a popular beverage in China and is well documented to have anti-oxidation and anticancer effects. EGCG, the most abundant and bioactive ingredient in green tea, is widely served in healthcare products. In this study, we investigated the anti-proliferation and anti-migration effects of EGCG in colorectal cancer *in vitro* to provide scientific evidence of EGCG for future application in colon-cancer prevention and treatment as drug supplements or adjuvant therapy.

In the present study, we found that treatment with EGCG resulted in a dose-dependent inhibition of the cell viability of colorectal-cancer SW480, SW620, and LS411N cells. To determine whether the anti-proliferative effect of EGCG was associated with apoptosis induction, Annexin V-FITC/PI double-staining and JC-1-staining assays were employed. The results showed that EGCG induced apoptosis in SW480, SW620, and LS411N cells in a dose-dependent manner. The findings were in line with the effect of EGCG on colorectal-cancer HT-29 cells, which inhibited cell proliferation in a dose-dependent manner, by inhibiting Akt, ERK1/2, or alternative p38MAPK activity [20]. EGCG was also demonstrated to be effective in inducing apoptosis in colorectal-cancer DLD-1 cells and showed a remarkably synergistic enhancement of growth inhibition and apoptosis when combined with resveratrol and 5-fluorouracil [21]. JC-1-staining results showed that EGCG induced disruption of the mitochondrial-membrane potential in SW480, SW620, and LS411N cells in a dose-dependent manner, indicating that EGCG induced apoptosis partly through the mitochondria pathway. The results were in line with previous findings that EGCG inhibited the growth of salivary adenoid cystic carcinoma cells via the EGFR/Erk signal-transduction pathway and the mitochondria pathway [22]. Apart from the anti-proliferation and apoptosis-induction effects, EGCG was also found to be effective at inhibiting SW480-cell migration in a dose-dependent manner as assessed by wound-healing and transwell-migration assays. The results were comparable to those of previous reports that EGCG could reduce the migration of SW620 cells [23]. EGCG was also found to be effective in the inhibition of proliferation and migration in human breast-cancer MCF-7 cells [24] and bladder-cancer T24 cells [25]. Moreover, the selected doses of EGCG for migration assays were non-cytotoxic doses (≤40 μg/mL) and the cell viability was >80%. These non-cytotoxic doses of EGCG showed remarkable anti-migration effects on SW480 cells. This means that EGCG could be added as an effective supplement for colon-cancer prevention and treatment.

To gain insight into the underlying mechanism of EGCG-induced apoptosis and migration inhibition, several proteins were tested, including Caspases-3, PARP, STAT3, p-STAT3, Bcl-2, Bim, Bak, MCL-1, E-cadherin, and Vimentin. EGCG was effective in the activation of Caspases-3 and PARP in colorectal-cancer cells, indicating that the anti-proliferative effect of EGCG was associated with apoptosis induction. The results were also in line with the data from Annexin V-FITC/PI double-staining and JC-1-staining assays that EGCG induced apoptosis and the disruption of mitochondrial-membrane potential in SW480, SW620, and LS411N cells in a dose-dependent manner.

STAT3 is a membrane-receptor-mediated nuclear transcription factor that ubiquitously exists in a dimerized form; once STAT3 is activated, it converts into p-STAT3 and activates the transcription of target genes downstream, such as Bcl-2 family members, MCL-1, etc. [26–27]. We found that treatment with EGCG resulted in a significant decrease in both STAT3 and p-STAT3, and suppression of anti-apoptotic proteins Bcl-2, Bim, and MCL-1. The result was in agreement with Yuan’s finding that EGCG treatment resulted in alteration of AKT/STAT3 signaling and downregulation of Bcl-2 in oral-cancer CAR cells [28]. A similar finding that EGCG induced a drastic decrease in the phosphorylation of STAT3 and led to downregulation of the target gene products of STAT3, such as Bcl-2, VEGF, MCL-1, and cyclin D1, was also found in neck-squamous-cell-carcinoma HNSCC cells [29]. In addition, we demonstrated that EGCG treatment in SW480, SW620, and LS411N cells resulted in a significant increase in the transition marker E-cadherin and the expression of Vimentin was significantly inhibited, indicating the anti-migration effects of EGCG. The results were consistent with the finding from wound-healing and transwell-migration assays that EGCG inhibits SW480-cell migration in a dose-dependent manner. The data were also comparable to Rieger-Christ’s finding that EGCG decreased the migratory potential of bladder-carcinoma cells with concomitant activation of MAPK and STAT3 and downregulation of N-cadherin [30]. A previous report from Li revealed that EGCG suppressed epithelial–mesenchymal transition and migration by blocking TGF-β/Smad-signaling pathways and downregulation of Vimentin in anaplastic-thyroid-carcinoma (ATC) 8505 C cells [31]. Treatment with EGCG in SW480, SW620, and LS411N cells resulted in the inhibition of STAT3, which might regulate downstream gene products, such as Caspase-3, Bcl-2, MCL-1, E-cadherin, and Vimentin, and at last contribute to the inhibition of migration and induction of apoptosis.

In order to confirm the importance of STAT3 in EGCG-induced proliferation and migration inhibition, the cell viability and migration were assessed when STAT3 was inhibited, and the STAT3 mRNA expression and promoter activity were also evaluated after treatment with EGCG. When STAT3 was inhibited, EGCG showed no obvious effect on SW480-cell proliferation and migration, indicating the important role that STAT3 plays in EGCG-induced inhibition. Furthermore, we found that EGCG suppressed STAT3 transcription and inhibited STAT3-promoter activity, suggesting that the suppression effect of EGCG on STAT3-promoter activity may be responsible for the downregulation of STAT3 mRNA expression. The result was in agreement with previous reports showing that EGCG treatment results in a drastic decrease in STAT3 activity in gastric cancer [32]; EGCG suppressed the phosphorylation of STAT3 in neck squamous-cell carcinoma [25]. However, these reports failed to elaborate on the underlying mechanism of how EGCG regulates STAT3. Our results have clearly demonstrated that EGCG decreases STAT3 expression in both protein and mRNA levels, and EGCG inhibits STAT3 protein more strongly than p-STAT3, indicating that the suppression of p-STAT3 might be caused by the inhibition of STAT3. Besides, we present first evidence that EGCG treatment results in the suppression of STAT3-promoter activity and STAT3 mRNA expression. This means that EGCG was not only effective in the transcription of STAT3 via the inhibition of promoter activity, but also affected the phosphorylation of STAT3 at the translation level, thus contributing to the suppression of STAT3. This revelation sheds light on the underlying mechanisms of EGCG on proliferation and migration inhibition in colon cancer, and provides clear directions for cancer treatment and drug combinations. In our study, the primary human colorectal-cancer SW480 cells and metastatic human colorectal-cancer SW620 and LS411N cells were used. We aim to investigate the anti-proliferation and anti-migration effects of EGCG in primary and metastatic colon-cancer cells, and compare the efficacy and sensitivity among the three cell lines. All the results from MTT, Annexin V-FITC/PI staining, JC-1 staining, scratch-wound healing, transwell-migration assay, and Western blot showed that EGCG was effective in all of the three colorectal-cancer cell lines with similar findings. The only difference is that SW480 cells were more sensitive to EGCG when compared to metastatic SW620 and LS411N cells, and the possible reason might be that SW480 cells are primary colon-cancer cells with less metastatic ability. The results demonstrated that EGCG was not only effective in primary human colorectal cancer, but also plays an important role in metastatic human colorectal cancer.

In summary, our results present the first evidence of the anti-proliferation and anti-migration effects of EGCG against colorectal-cancer SW480, SW620, and LS411N cells by downregulating the expression of STAT3. This revelation sheds light on the underlying mechanisms of EGCG on tumor suppression in colon cancer and suggests that EGCG might be an effective and natural supplement for cancer treatment and health protection. However, more detailed molecular mechanisms, such as the genomic and proteomic responses underlying the EGCG-induced colorectal-cancer-cell apoptosis, how EGCG suppresses STAT3 promoters, anti-metastasis, and anti-angiogenesis, remain to be elucidated. In addition, more investigations should be included to elaborate the efficacy in animal and human subjects with colon cancer and determine the clinical efficacy and safety of EGCG. Our observation holds promise for further studies to examine the efficacy of EGCG and develop EGCG as a potential anticancer supplement against colon cancer.

## Authors’ contributions

K.W.L. provided the idea and wrote the paper; K.W.L., J.X., B.H.C., and H.C.G. were involved in the cell-culture, flow-cytometry experiments, Western blot, transwell-migration assay, and luciferase-reporter-activity assay; L.W.F. and X.L.L. designed the work and revised the paper. All authors reviewed and approved the final manuscript.

## Funding

This work was supported by grants from Shenzhen Longhua District Science and Technology Innovation Breau [201801 to K.W.L.], Shenzhen Municipal Science and Technology program of China [JCYJ20160425100840929 to K.W.L.], and the Natural Science Foundation of Guangdong province [2019A1515011009 to H.C.G.].
